# Research Note: Evaluation of a precision-fed rooster assay for determination of phytic acid disappearance in feedstuffs

**DOI:** 10.1016/j.psj.2022.102356

**Published:** 2022-11-19

**Authors:** B.W. Parsons, P.L. Utterback, C.M. Parsons, S.J. Rochell, J.L. Emmert

**Affiliations:** ⁎ Department of Poultry Science, University of Arkansas at Fayetteville, AR 72701, USA; † Department of Animal Sciences, University of Illinois at Urbana-Champaign, IL 61801, USA

**Keywords:** feed ingredients, phosphorus, phytase, phytic acid, precision-fed rooster

## Abstract

The objective of this study was to evaluate a precision-fed rooster assay that is suitable for determination of phytic acid (myo-inositol 1,2,3,4,5,6-hexakis; InsP_6_) disappearance in plant-based feed ingredients. A 48-h precision-fed rooster assay was used to measure InsP_6_ disappearance using conventional White Leghorn roosters. A minimum of 4 individually-caged roosters per treatment were fasted for 26 h prior to crop intubation with 15 to 30 g of sample, and excreta were quantitatively collected for 48 h. Soybean meal, soybean hulls, canola meal, conventional distillers dried grains with solubles (**DDGS**), palm kernel meal (**PKM**), and wheat bran were evaluated in Experiment 1, whereas wheat middlings (**WM**) and rice bran (**RB**) were evaluated without and with 1,000 and 1,800 U/kg phytase in Experiment 2. Data from Experiment 1 were subjected to a one-way ANOVA for a completely randomized design, while data from Experiment 2 were subjected to two-way ANOVA for a 2 × 3 factorial arrangement of treatments. In Experiment 1, InsP_6_ disappearance ranged from 3 to 95% among all ingredients. The InsP_6_ disappearance for conventional DDGS (95%) was the highest (*P* < 0.05), wheat bran and soybean hulls were intermediate (47–48%), PKM was low (24%), and soybean meal and canola meal were very low (3–5%). In Experiment 2, there was a significant ingredient × phytase interaction (*P* < 0.05). Phytase inclusion at both 1,000 and 1,800 U/kg resulted in a significant improvement (*P* < 0.05) in InsP_6_ disappearance for RB; however, only the addition of 1,800 U/kg resulted in an increase in InsP_6_ disappearance for WM. The addition of 1,800 U/kg phytase increased the InsP_6_ disappearance from 58 to 74% for WM and from 26 to 53% for RB. These results suggest the precision-fed rooster assay can be used to evaluate phytic acid disappearance in plant-based feed ingredients and the assay was able to detect a significant effect of 1,800 U/kg of exogenous phytase on phytic acid disappearance for WM and RB.

## INTRODUCTION

The precision-fed rooster assay has been used extensively to determine ME and amino acid digestibility in feed ingredients (Sibbald, 1976; Parsons et al., 1981). Munoz et al. (2018) utilized the precision-fed rooster assay to measure total tract P retention in feed ingredients; however, it was concluded that due to the high endogenous P excretion by the roosters and the low P requirement of the roosters, which necessitates feeding low non-phytate P levels to prevent absorbed dietary P from being excreted in the urine, this assay would not be suitable to evaluate P availability for most feed ingredients. It has been shown that 50% or more of the P in many plant-based ingredients is bound in phytic acid (Tahir et al., 2012). In order for phytate P to be utilized by the bird, it must first be hydrolyzed and released from phytate. The amount of digestible P in diets has been found to be influenced by the amount of phytic acid that is hydrolyzed (Rodehutscord, 2016). In particular, data compiled from several studies by Rodehutscord (2016) indicated that for every gram of phytic acid (myo-inositol 1,2,3,4,5,6-hexakis; **InsP_6_**) that disappeared, the amount of P digested increased by 0.78 g. The latter suggests that InsP_6_ disappearance may have potential for predicting P digestibility; therefore, a precision-fed rooster assay in which phytic acid disappearance in feed ingredients is measured rather than total tract P retention, as done in the study by Munoz et al. (2018), has potential for predicting or estimating P digestibility for routine evaluation of feed ingredients. To our knowledge, no previous studies have been published on evaluating the precision-fed rooster assay for measuring phytic acid disappearance in feed ingredients; therefore, the objective of this study was to evaluate the use of a precision-fed rooster assay to determine phytic acid disappearance in feed ingredients with or without the use of exogenous phytase.

## MATERIALS AND METHODS

The protocol for this study was reviewed and approved by the Institutional Animal Care and Use Committee at the University of Illinois (protocol number 20131).

### Ingredients and Analysis

A total of 8 feed ingredients were evaluated in this study. In Experiment 1, dehulled solvent extracted soybean meal, soybean hulls, conventional distillers dried grains with solubles (**DDGS**), palm kernel meal, and wheat bran were evaluated. Wheat middlings and rice bran were evaluated in Experiment 2. All samples were obtained from commercial companies within the United States, excluding palm kernel meal which was obtained from Indonesia. The phytase utilized in Experiment 2 was supplied by Danisco Animal Nutrition/IFF (Wilmington, DE). Analyses were conducted by the Agricultural Experiment Station Chemical Laboratory (University of Missouri, Columbia, MO) to determine CP by measuring N content via combustion (Method 990.03; AOAC International, 2007), crude fat (Method 920.39 A; AOAC International, 2007), neutral detergent fiber (**NDF**) (Method 2002.04; AOAC International, 2007), and Ca and P via inductively coupled plasma optical emission spectrometry (Method 958.01 A, B, and D; AOAC International, 2007). Analyses were conducted by Eurofins (Des Moines, IA) to determine InsP_6_ concentration (Ellis et al, 1977). Phytase activity (U/kg) in the feed ingredients was determined by AB Vista (Plantation, FL) by ELISA (ESC, Standard Analytical Method, SAM099; AB Vista). Dry matter content of samples was determined at the University of Illinois (Method 930.15; AOAC International, 2007).

### Diets and Experimental Design

Two experiments were conducted with conventional adult Single Comb White Leghorn roosters using the precision-fed rooster assay. Roosters were fasted for 26 h prior to being precision-fed (crop intubated) between 15 and 30 g of sample. Ingredients were fed as received (no additional grinding). Feed was weighed into individual beakers and the amount of each ingredient that was precision-fed was adjusted among ingredients to achieve a similar volume of feed due to variation in the density of feedstuffs evaluated herein and in attempt to prevent crop impaction. The addition of phytase to diets in Experiment 2 was conducted by mixing a larger batch of diet using a tabletop mixer because of the small amount of phytase being added. There were 5 replications of 1 individually caged rooster per treatment in Experiment 1, except for palm kernel meal which had 4 replications of 1 individually caged rooster because 1 rooster exhibited crop compaction which prevented an accurate quantitative excreta collection. There were 4 replications of 1 individually caged rooster per treatment in Experiment 2. All birds were provided water ad libitum. After feeding, a tray was place beneath each cage and excreta were collected after 48 h. In addition, 5 roosters were fasted continually in Experiment 1 to determine if there was any endogenous phytic acid being excreted, and excreta were also collected for 48 h for this assessment. After collection, excreta were stored in a freezer prior to lyophilization, after which samples were weighed, ground, and analyzed for InsP_6_ content. The InsP_6_ disappearance was then calculated using the equation shown below:

$$\text{Ins}P_{6}\text{disappearance}\left(\%\right)=\left[\left(\text{Ins}P_{6}\text{intake}-\text{Ins}P_{6}\text{excreted}\right)/\text{Ins}P_{6}\text{intake}\right]\times 100$$

where InsP_6_ intake (g) = diet intake (g) × (InsP_6_ in diet (%)/100); InsP_6_ excreted (g) = excreta output (g) × (InsP_6_ in excreta (%)/100).

### Statistical Analysis

The Proc GLM procedure in SAS (SAS Institute; Cary, NC) was used to analyze data from Experiment 1 using a one-way ANOVA procedure for a completely randomized design. Data in Experiment 2 were subjected to a 2-way ANOVA for a 2 × 3 factorial arrangement of treatments with ingredient (wheat middlings and rice bran) and phytase (0, 1,000, and 1,800 U/kg) as main effect variables. Pairwise treatment comparisons were conducted following the ANOVA procedure for both experiments using Fisher's least significant difference test. The probability level of *P* < 0.05 was used to determine significant differences for all comparisons.

## RESULTS AND DISCUSSION

The analyzed nutrient composition of feedstuffs on a DM basis is presented in Table 1. The Ca, P, and InsP_6_ content of feedstuffs varied substantially. The InsP_6_ content ranged from 0.31% in soybean hulls to 5.77% in rice bran. Total P and phytate P (InsP_6_-P) values in Table 1 are in good agreement with the study by Leske and Coon (1999), where the phytate P content of soybean meal, canola meal, wheat middlings, and rice bran in the present study were 0.43%, 0.90%, 1.13%, and 1.62%, respectively, which were similar to 0.40%, 0.82%, 1.19%, and 1.62% (DM basis), respectively, reported by those authors. There was, however, considerable variation between the phytic acid content reported for DDGS in the present study (0.66%) compared with the value of 0.26% reported by Tahir et al. (2012). Some of this variability may be attributed to differences in the analytical methods used to determine the phytate P content, as well as differences in the processing methods used to produce DDGS.

**Table 1:** Analyzed nutrient composition of feed ingredients (%).^1^

	DM	CP	Crude fat	NDF^2^	Ca	P	InsP_6_^3^	InsP_6_-P^4^	Phytase activity (U/kg DM)
Soybean meal	88.2	52.4	1.0	7.4	0.32	0.71	1.52	0.43	<50
Soybean hulls	89.3	16.9	4.7	57.4	0.59	0.22	0.31	0.09	540
Canola meal	87.9	43.1	3.4	26.8	0.73	1.18	3.21	0.90	<50
Conventional DDGS^5^	88.0	33.1	7.1	38.7	0.30	1.14	2.35	0.66	1,807
Palm kernel meal	96.6	17.2	6.1	67.3	0.55	0.87	1.67	0.47	1,139
Wheat bran	89.3	16.5	2.9	51.1	0.11	1.50	5.25	1.48	1,982
Wheat middlings	87.5	15.6	3.5	39.0	0.12	1.19	4.01	1.13	1,520
Rice bran	92.0	15.9	17.1	15.6	0.48	1.96	5.77	1.62	55

1 Values are expressed on a DM basis, excluding DM which is expressed on an as-fed basis.

2 NDF = neutral detergent fiber.

3 InsP_6_ = myo-inositol 1,2,3,4,5,6-hexakis, phytic acid.

4 InsP_6_-P = InsP_6_ × 0.28, which is the estimate of phytate bound P.

5 Conventional DDGS = conventional distillers dried grains with solubles.

Phytase activity varied greatly among feedstuffs (Table 1). The phytase activities in soybean meal, canola meal, and rice bran were 55 U/kg DM or less, whereas soybean hulls contained 540 U/kg, palm kernel meal contained 1,139 U/kg, and conventional DDGS, wheat bran, and wheat middlings contained more than 1,500 U/kg. Corn has been reported to contain a low phytase activity of 143 U/kg DM (Rodehutscord et al., 2016); therefore, the high phytase activity in conventional DDGS is likely due to the addition of exogenous phytase during the liquefaction and simultaneous saccharification and fermentation steps in dry grind corn processing. Unlike corn, wheat has been reported to contain higher levels of phytase activity. Rodehutscord et al. (2016) evaluated 29 samples of wheat and reported a mean phytase activity of 1,850 U/kg DM. The phytase activities reported for wheat samples by Rodehutscord et al. (2016) are comparable with the phytase activity of the wheat co-products evaluated herein, where wheat bran and wheat middlings contained 1,982 U/kg and 1,520 U/kg, respectively.

No detectible phytic acid was found in the excreta of the roosters that were fasted continually in Experiment 1 (data not shown). The InsP_6_ disappearance of feedstuffs is presented in Table 2. In Experiment 1, InsP_6_ disappearance was the lowest (*P* < 0.05) for canola meal and soybean meal (3.2% and 5.2%, respectively), low for palm kernel meal (23.6%), intermediate for wheat bran and soybean hulls (47.2% and 47.6%, respectively), and high for conventional DDGS (95.3%). Leske and Coon (1999) reported higher InsP_6_ disappearances for soybean meal and canola meal in 3-week-old broilers, which were 35% and 37%, respectively. This discrepancy between studies may be partially explained by the differences in the bioassay used, where broilers provided diets ad libitum may have elevated phytase activity in the small intestine or increased microbial break down of phytic acid in the ceca compared with precision-fed roosters. The extent of InsP_6_ disappearance of feedstuffs (Table 2) was generally in good agreement with phytase activity (Table 1). Both canola meal and soybean meal had negligible InsP_6_ disappearance, which was consistent with phytase activity that was below the detection threshold, while the higher InsP_6_ disappearance in wheat bran and conventional DDGS were consistent with high phytase activities (1,982 and 1,807 U/kg DM, respectively) in those ingredients. Despite the slightly higher phytase activity in wheat bran compared with conventional DDGS, wheat bran had a lower (*P* < 0.05) InsP_6_ disappearance. This may be due to the higher phytic acid intake of birds fed wheat bran, which would require a greater amount of phytase to break down the larger amount of substrate consumed, where roosters precision-fed wheat bran consumed 0.70 g of InsP_6_ compared with 0.52 g for roosters fed DDGS. Soybean hulls exhibited similar InsP_6_ disappearance to wheat bran despite having substantially less phytase activity. This is likely due to the low InsP_6_ intake of birds fed soybean hulls (0.05%), in which a small amount of phytic acid breakdown in soybean hulls would result in a much larger percentage increase in InsP_6_ disappearance compared with wheat bran. The high phytase activity in palm kernel meal (1,139 U/kg DM) is also in contrast to the low InsP_6_ disappearance of 23.6% in Experiment 1. Although the cause for this remains to be elucidated, the pH and retention time in the gastrointestinal tract as well as the high fiber content in palm kernel meal may have resulted in altered conditions in vivo compared with the in vitro phytase analysis, thus altering phytase activity.

**Table 2:** Phytic acid intake and disappearance in precision-fed roosters.

	Treatment	Feed intake (g)	InsP_6_ intake^1^ (g)	InsP_6_ disappearance^1^^,^^2^ (%)	Pooled SD^3^
Experiment 1	Soybean meal	25.0	0.34	5.2^d^	5.07
	Soybean hulls	20.0	0.05	47.6^b^	
	Canola meal	25.0	0.71	3.2^d^	
	Conventional DDGS^1^	25.0	0.52	95.3^a^	
	Palm kernel meal	30.0	0.48	23.6^c^	
	Wheat bran	15.0	0.70	47.2^b^	
Experiment 2^4^	Wheat middlings	20.0	0.70	58.2^b^	7.08
	Wheat middlings + 1,000 U/kg^5^	20.0	0.70	57.4^b^	
	Wheat middlings + 1,800 U/kg^5^	20.0	0.70	74.2^a^	
	Rice bran	20.0	1.06	25.8^d^	
	Rice bran + 1,000 U/kg^5^	20.0	1.06	44.4^c^	
	Rice bran + 1,800 U/kg^5^	20.0	1.06	53.4^bc^	

a-d Means within an experiment and column with no common superscript are significantly different (*P* < 0.05).

1 InsP_6_ = myo-inositol 1,2,3,4,5,6-hexakis, phytic acid; conventional DDGS = conventional distillers dried grains with solubles.

2 Values are means of 5 roosters in Experiment 1, excluding palm kernel meal which is a mean of 4 roosters, and 4 roosters in Experiment 2.

3 Pooled SD of InsP_6_ disappearance values.

4 Significant effect of diet, phytase, and diet × phytase (*P* < 0.05).

5 U/kg = units of phytase per kg of diet.

The ability to accurately predict P availability by measuring InsP_6_ disappearance in precision-fed roosters warrants further research. The samples of soybean meal and conventional DDGS evaluated in the present study were previously analyzed for P digestibility and relative bioavailability in broiler chickens in our laboratory. The ileal P digestibility and relative bioavailability values (based on tibia bone ash) of the soybean meal used in the present study were 55% and 27%, respectively, E. M. Ahasic (University of Illinois, Urbana, IL, personal communication). As stated earlier, data compiled from several studies by Rodehutschord (2016) suggests that for every gram of InsP_6_-P that disappears from the digestive tract, the P digested increases by 0.78 g. If this conversion factor is used and non-phytate P is assumed to be 100% digestible or bioavailable, then digestibility of the P in the soybean meal predicted from the experimentally determined InsP_6_ disappearance herein would be 35%. This P digestibility estimation of 35% in soybean meal based on InsP_6_ disappearance is similar to the previously determined P relative bioavailability of 27%, but is numerically lower than the previously determined ileal P digestibility of 55%. Using the same calculations and assumptions as above, the predicted P digestibility in conventional DDGS based on InsP_6_ disappearance (Table 2) would be 87%. This value is higher than the previously determined ileal P digestibility and relative bioavailability bone ash values of 63% and 61%, respectively (B. Parsons, unpublished data). The higher predicted P digestibility value in DDGS based on InsP_6_ disappearance compared with the previously determined P digestibility and relative bioavailability values may potentially be due to the overestimation of P digestibility in the non-phytate phosphorus fraction. Estimated total tract P retention of the non-phytate P fraction by Leske and Coon (1999) and Leske and Coon (2002) demonstrates that the assumption that 100% of the non-phytate P fraction is retained is likely incorrect and may more accurately range from 60% to 80%. Further evaluation of feedstuffs is needed to better characterize the relationship between InsP_6_ disappearance in precision-fed roosters and P digestibility and bioavailability in broilers.

There was a significant interaction (*P* < 0.05) between feedstuff and exogenous phytase activity in Experiment 2 (Table 2). Although the addition of both 1,000 and 1,800 U/kg phytase increased InsP_6_ disappearance in rice bran, only the addition of 1,800 U/kg phytase increased phytic acid disappearance in wheat middlings. The InsP_6_ disappearance in wheat middlings without exogenous phytase was 58%, which was increased to 74% by the addition of 1,800 U/kg phytase. The InsP_6_ disappearance of rice bran without the addition of exogenous phytase was 26%, which was increased to 44% and 53% with 1,000 U/kg and 1,800 U/kg of phytase, respectively. The lower InsP_6_ disappearance in rice bran compared with wheat middlings without the addition of exogenous phytase may be explained by the higher endogenous phytase activity in wheat middlings. The cause for the lack of response to the addition of 1,000 U/kg in wheat middlings is unknown and may have been at least partially associated with the higher InsP_6_ disappearance in wheat middlings compared with rice bran with 0 U/kg of exogenous phytase; more research is needed to confirm the InsP_6_ disappearance reported herein for wheat middlings with exogenous phytase using the precision-fed rooster assay. Leske and Coon (1999) reported InsP_6_ disappearance in broilers fed rice bran and wheat middlings to be 33% and 29%, respectively. The disappearance of InsP_6_ was increased for both ingredients to 48% and 52%, respectively, when 600 U/kg of phytase were added. Leske and Coon (1999) reported similar InsP_6_ disappearance in rice bran and similar effects of exogenous phytase on InsP_6_ disappearance compared with the present study; however, the InsP_6_ disappearance in wheat middlings without the use of exogenous phytase was lower in the study by Leske and Coon (1999). The difference in InsP_6_ disappearance for wheat middlings between studies may be due to differences in endogenous phytase activity of the samples that were evaluated.

Although utilization of the precision-fed rooster assay to determine phytic acid disappearance does not directly determine P digestibility or relative bioavailability, this assay appears to circumvent many of the confounding factors observed by Munoz et al. (2018) when utilizing the precision-fed rooster assay to directly measure total tract P retention. For instance, as mentioned earlier, there was no InsP_6_ detected in excreta from fasted roosters (data not shown), whereas in the study by Munoz et al. (2018), the basal endogenous P losses in the roosters were substantial (108 mg per bird). These authors also concluded that when utilizing the precision-fed rooster assay to evaluate P retention, the non-phytate P intake must be restricted to 50 to 70 mg, which often requires feeding low levels of feedstuffs. This is due to the low P requirement of the roosters, in which feeding levels of bioavailable P above the animal's requirement causes excess absorbed P to be excreted in the urine, thus decreasing the P retention values for the feedstuff. Measuring InsP_6_ disappearance in precision-fed roosters eliminates the need to feed low levels of non-phytate P and avoids the confounding factor of high endogenous P losses, making this a more accurate and widely applicable assay than measuring P retention when using the precision-fed rooster assay for routine evaluation of plant-based feed ingredients.

Overall, data from these experiments indicate the precision-fed rooster assay can be used to measure phytic acid disappearance for a wide variety of plant-based feedstuffs. Moreover, the assay may possess some capacity to detect the breakdown of phytic acid by exogenous phytase in the digestive tract since a significant increase in phytic acid disappearance was observed by feeding 1800 U/kg of exogenous phytase for wheat middlings and rice bran. Further work is needed to compare InsP_6_ disappearance with disappearance of other phytic acid isomers and to fully elucidate the relationship between InsP_6_ disappearance in precision-fed roosters and digestibility or bioavailability of P in feedstuffs for poultry.

## DISCLOSURES

The authors declare that they have no known competing financial interests or personal relationships that could have appeared to influence the work reported in this paper.
